# Synthesis and Application of Porous Carbon Nanomaterials from Pomelo Peels: A Review

**DOI:** 10.3390/molecules28114429

**Published:** 2023-05-30

**Authors:** Zixuan Liu, Qizheng Yang, Lei Cao, Shuo Li, Xiangchen Zeng, Wenbo Zhou, Cheng Zhang

**Affiliations:** College of Engineering, Nanjing Agricultural University, Nanjing 210031, China; 9213011016@stu.njau.edu.cn (Z.L.); 9203011726@stu.njau.edu.cn (Q.Y.); 9203011801@stu.njau.edu.cn (L.C.); 9203011713@stu.njau.edu.cn (S.L.); 18942449245@163.com (X.Z.); 15951661171@163.com (W.Z.)

**Keywords:** pomelo peels, porous carbon, nanomaterials, biochar

## Abstract

Advanced carbon nanomaterials have been widely applied in various fields such as microelectronics, energy storage, catalysis, adsorption, biomedical engineering, and material strengthening. With the increasing demand for porous carbon nanomaterials, many studies have explored obtaining porous carbon nanomaterials from biomass, which is highly abundant. Pomelo peel, a type of biomass rich in cellulose and lignin, has been widely upgraded into porous carbon nanomaterials with large yield and significant applications. Here, we systematically review the recent progress in pyrolysis, activation, and applications of synthesizing porous carbon nanomaterials from waste pomelo peels. Moreover, we provide a perspective on the remaining challenges and potential future research directions.

## 1. Introduction

Advanced carbon nanomaterials, such as biochar, graphene, and carbon nanotube, have wide applications in heavy-metal ion absorption [1], energy storage [2], and sensing owing to their excellent properties, such as high specific surface area (SSA) [3], high electrical conductivity [4], and so on. Various methods have been developed to synthesize advanced carbon materials from a wide range of raw sources. For example, chemical vapor deposition (CVD) method has been employed to fabricate high quality graphene from methane gas [5]. However, these methods are complicated and the used raw sources are rare or expensive, which hinders the large-scale production and commercialization of advanced carbon materials.

Biomass is an intriguing raw source for advanced porous carbon nanomaterials through simple pyrolysis [6]. Generally, extraction advanced porous carbon nanomaterials from biomass have two advantages. First, the cost to obtain the advanced porous carbon nanomaterials can be largely reduced. Biomass has a variety of sources, from crop straws, shuck, leaves, fruit peels, and microorganisms [7] to chitin [8], which are low-value by-productions from agriculture, industry, and daily life. Second, the environmental pollution resulting from biomass can be relieved [9]. Biomass is typically discarded, resulting in environmental pollution, especially a large amount of carbon dioxide (CO_2_), which is emitted into the atmosphere and aggravates the greenhouse effect.

Amongst diverse biomass sources, pomelo peel (PP) has attracted increasing interest. First, it has large production. Take China, the major producer and consumer country, as an example. In recent years, the produce of pomelos in China is as high as 5 million tons and 30~50% (*w*/*w*) of pomelo is peel [10]. If we take reasonable ways to process and use PP, we will gain high rewards. Second, it has a highly porous structure. The performance of advanced carbon materials prepared from biomass is closely related to the structural characteristics of biomass. Biomass materials with a loose and porous structure can show excellent electrochemical performance after carbonization and activation [11].

**Figure 1:**
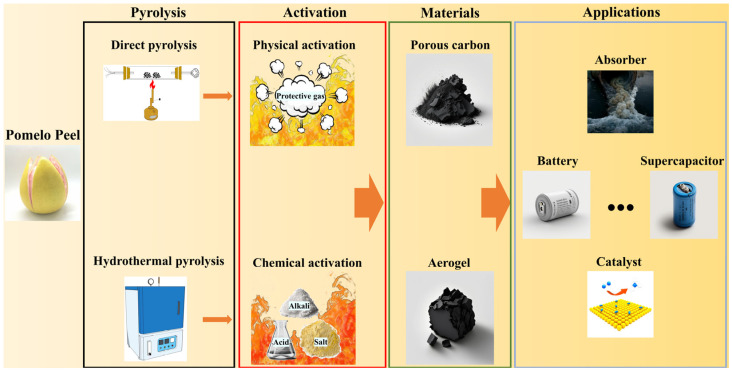
Synthesis of porous carbon nanomaterials from waste pomelo peels.

As shown in Figure 1, in this review, we will summarize the process of upgrading PP into porous carbon nanomaterials through direct pyrolysis and hydrothermal pyrolysis. Afterwards, we focus on the activation step, including physical activation, chemical activation, and their combination, which directly affects the properties of the produced porous carbon nanomaterials. Subsequently, the applications of the porous carbon nanomaterials obtained from pyrolyzed PP were summarized, such as adsorbers, batteries, supercapacitors, and catalysts. Finally, the discussions are concluded with an overview of the challenges and possible new ways to upgrade PP into porous carbon nanomaterials.

## 2. Pyrolysis

Currently, there are two primary methods for pyrolyzing PP to porous carbon nanomaterials: direct pyrolysis and hydrothermal pyrolysis. The former is a conventional and uncomplicated technique, while the latter is an emerging technique with its capacity for processing PP continually being developed [12]. The two techniques have different methods of operation and produce porous carbon nanomaterials with different properties (Table 1), and each has its own advantages and disadvantages in practical applications.

### 2.1. Direct Pyrolysis

Direct pyrolysis is a commonly used method for converting PP into porous carbon nanomaterials. It mainly takes advantage of the instability of organic matter and retains the solid phase porous carbon nanomaterials through high temperature decomposition under anaerobic or anoxic conditions. With the help of direct pyrolysis technology, PP can be processed to obtain porous carbon. It is a type of carbon-based material with a diverse structure, light weight, large surface area, and rich surface chemistry, making it highly useful for various industrial applications [40]. Porous carbon can be produced on a large scale by direct pyrolysis of PP.

In general, direct pyrolysis of PP to porous carbon nanomaterials can be divided into three steps [16]. The first step is to pre-treat the PP, including washing, to remove impurities, dicing the crop to a size suitable for further processing, and drying it to reduce the excess moisture. The second step is to carbonize the pre-treated PP pieces, which is usually performed in an atmosphere of inert gas. The third step is to activate and dry the carbonized powder, where the activation step is discussed in detail in the next section. 

**Figure 2:**
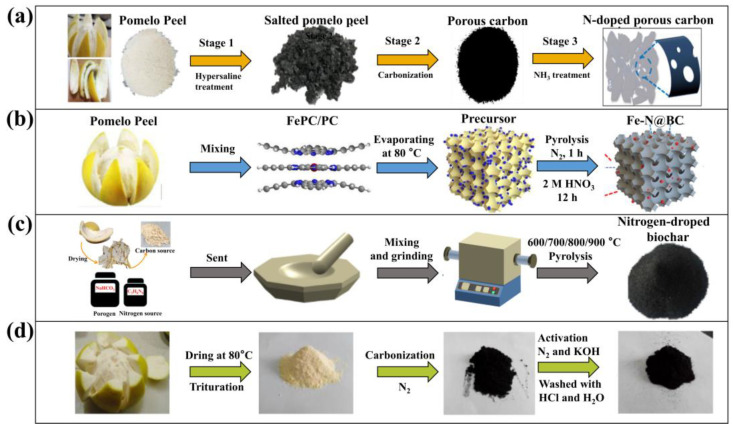
Direct pyrolysis of PP to porous carbon nanomaterials for various applications. (**a**) Nitrogen-doped porous carbon with electrocatalytic activity [19]. Reproduced with permission, Copyright 2018, Elsevier, Amsterdam, The Netherlands. (**b**) Fe-N@BC as catalyst for the reduction of hexavalent chromium [41]. Reproduced with permission, Copyright 2022, Elsevier. (**c**) N-doped biochar as peroxymonosulfate activator [42]. Reproduced with permission, Copyright 2022, Elsevier. (**d**) Porous carbon as absorber for methyl orange [16]. Reproduced with permission, Copyright 2016, Elsevier.

Wang et al. prepared the PP-derived porous carbon through a three-stage direct pyrolysis process (Figure 2a) [19]. In the first stage, dried PP was treated with hypersaline to initially expand the pores, resulting in a carbon precursor with abundant pores. In the second stage, the precursor was pre-carbonized at 400 °C for 3 h in an ammonia atmosphere, and then carbonized at 700 °C for 2 h to obtain the porous carbon. In the third stage, potassium hydroxide was used as a catalyst, and the precursor was activated for 2 h in an argon atmosphere, followed by high-temperature heating in a nitrogen atmosphere to dope nitrogen atoms. The resulting porous carbon material not only exhibits abundant pores and ultra-high SSA but also possesses the catalytic ability for redox reactions due to the abundant nitrogen doping. As the problem of Cr^6+^ pollution in water becomes increasingly severe, Yao et al. have proposed a biochar catalyst (BC) doped with iron and nitrogen (Figure 2b) [41]. Firstly, iron phthalocyanine (FePC) was mixed with PP. Then, the mixture was slowly dried at 80 °C. Finally, the mixture was carbonized at high temperature for 1 h under a nitrogen atmosphere and activated with HNO_3_ for 12 h to obtain Fe-N@BC. This material can efficiently catalyze the reduction of Cr^6+^ to the less toxic Cr^3+^. Dried pomelo peels can be co-ground with NaHCO_3_ and melamine, and then pyrolyzed at 600 °C for 2 h under a nitrogen atmosphere to obtain a highly nitrogen-doped biochar. This material can serve as an efficient catalyst for sulfamethoxazole (SMX) (Figure 2c) [42]. In particular, porous carbon nanomaterials that exhibit adsorption properties can be easily prepared using a simple and basic direct pyrolysis process (Figure 2d) [16]. Dried PP at 80 °C was carbonized for 1.5 h and 2.5 h at 450 °C and 800 °C, respectively, under a nitrogen atmosphere, and then activated using KOH in a nitrogen atmosphere. The resulting porous carbon exhibited significant adsorption properties for methyl orange and is cost-effective. Table 2 summarizes the preparation process of typical research of direct pyrolysis to prepare porous carbon.

The advantages of direct pyrolysis include simple operation and low cost for small-scale preparation. The porous carbon nanomaterials prepared by direct pyrolysis PP inevitably experience structural collapse due to high temperatures [47]. Similar phenomena have been explained through biomass pyrolysis studies, particularly those involving PP-like biomass. The pyrolysis of Populus euphratica wood enters the pore shrinkage stage after reaching 350 °C. As the temperature increases, the solid cell wall material collapses the cell lumen as some liquid-phase substances flow out of the cell wall [48]. In the temperature range of 600 °C to 900 °C, waste leather gradually undergoes micropore collapse and agglomeration into mesopores, resulting in a decrease in porosity [49]. The studies summarized in Table 2 show that direct pyrolysis of PP generally occurs at temperatures between 600 °C and 1000 °C, which is the temperature range at which cell wall exploration occurs and micropores aggregate into medium to large pores. It greatly reduces the electrochemical properties of the resulting porous carbon nanomaterials [50,51].

### 2.2. Hydrothermal Pyrolysis

Hydrothermal pyrolysis is a method to prepare porous carbon nanomaterials under moderate temperature but high-pressure conditions using water or mixed solution as solvents. Under this environment, lignin and cellulose of biomass are dehydrated and decarboxylated to form porous carbon nanomaterials [52]. Compared with direct pyrolysis, the hydrothermal pyrolysis does not require high temperature and can prepare a wider variety of materials including porous carbon and aerogel. Porous carbon is stable in shape, rich in porosity, and highly conductive. By slightly adjusting the process of hydrothermal pyrolysis, a lighter, more flexible, and almost non-conductive carbon aerogel can be obtained [53]. These two materials have different properties and can be used in different fields.

In most cases, hydrothermal pyrolysis of PP to obtain conventional porous carbon can be divided into four steps. The first step is similar to the direct pyrolysis, where the PP is initially washed with deionized water or a mixed solution and diced or crushed to make it suitable for the subsequent process. The second step is the high-pressure hydrothermal pyrolysis, in which the pre-treated PP mixed with a solvent and converted into carbon nanomaterials under certain temperature and high pressure conditions. The third step is to activate the precursors by physical means or chemical agents, which result in a larger SSA and a greater abundance of oxygen-containing functional groups. The last step is to freeze-dry the delaminated carbon nanomaterials, which usually takes up most of the time of the whole preparation process. 

**Figure 3:**
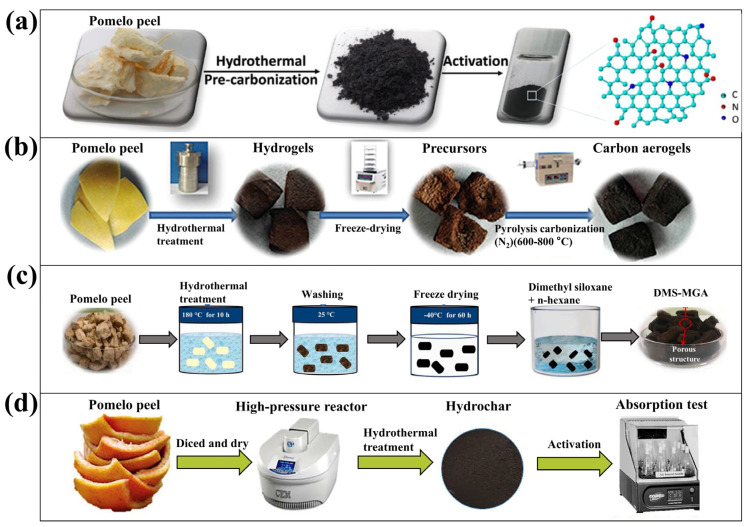
Hydrothermal pyrolysis PP to porous carbon nanomaterials for various applications. (**a**) Porous framework-like N-doped carbon (PFNC) with application as supercapacitor [33]. (Reproduced with permission, Copyright 2016, ACS). (**b**) Porous carbon with ability to adsorb Cu^2+^ [54]. (Reproduced with permission, Copyright 2017, Elsevier). (**c**) Biomass-based carbon aerogel to adsorb organics [23]. Reproduced with permission, Copyright 2017, Elsevier. (**d**) 3D porous superoleophilic/hydrophobicand carbon aerogel with oil adsorption capacity [55]. (Reproduced with permission, Copyright 2023, Elsevier).

Qu et al. combined porous carbon and plate-like NiO prepared from hydrothermally pyrolyzed PP to form an array-type supercapacitor (Figure 3a) [33]. The porous carbon was obtained through a two-step process. In the first step, the PP was chopped and dried, and then pre-carbonized at high pressure and temperature about 160 °C for 12 h, followed by carbonization at atmospheric pressure and 300 °C for 1 h to obtain pre-carbonized powder. The second step was mixing the pre-carbonized powder with KOH powder, and activating the mixture at 800 °C for 2 h under a nitrogen atmosphere. In this way, we can obtain porous framework-like N-doped carbon (PFNC), which exhibited a specific capacitance of 260 F g^−1^. Table 3 summarizes the preparation process of typical works of the hydrothermal method to prepare conventional porous carbon.

Conventional porous carbon prepared by hydrothermal pyrolysis processes more oxygen-containing functional groups and less impurities and ash compared with that prepared by direct pyrolysis [52]. However, it also consumes significantly more time to carbonize PP than direct pyrolysis.

As a complement to conventional hydrothermal pyrolysis, microwave-assisted hydrothermal pyrolysis is an efficient and highly promising technology for the preparation of conventional porous carbon [57]. This method uses microwaves to generate heat uniformly and rapidly by causing friction between the molecules of the raw material. Compared with direct pyrolysis and conventional hydrothermal pyrolysis, microwave-assisted pyrolysis of biomass produces higher solid. The solid products have a larger SSA and more regular pores [58]. With the assistance of microwaves, the high-pressure carbonization step in hydrothermal pyrolysis of PP can be completed within one hour, which is significantly shorter than the several hours required by conventional methods. By controlling the power of the microwaves, low-temperature microwave-assisted hydrothermal pyrolysis can achieve regular porosity with high energy efficiency [59], while high-temperature microwave-assisted hydrothermal pyrolysis yields materials with higher SSA [60,61]. Two-step low-temperature microwave-assisted hydrothermal heating was also developed (Figure 3b) [54]. The first step separates the water-soluble materials in PP and initially establishes a porous structure; the second step carries out a slightly higher temperature microwave-assisted hydrothermal heating of the solid residue obtained in the first step to complete the carbonization. Subsequently, the porous carbon material was subjected to Cu^2+^ adsorption experiments, and the results showed that the material was capable of spontaneously and efficiently adsorbing Cu^2+^.

Different from the conventional porous carbon, aerogel is an emerging porous nanomaterial, displaying characteristics of low density, high SSA, high dielectric strength, and tunable morphology. In particular, compared to traditional polymer aerogels, carbon aerogels produced from the pyrolysis of biomass have significant advantages in these aspects, and also have a strong electrical conductivity. Carbon aerogels are widely used in multiple fields such as biomedicine, aerospace, and implantable sensors [62]. However, the traditional method of extracting carbon aerogel precursors from the petroleum industry has the disadvantages of the depleting petroleum stocks and generating harmful by-products such as formaldehyde. Therefore, research has increasingly focused on the extraction of carbon aerogels from waste biomass [63].

Hydrothermal pyrolysis of PP to obtain aerogel can be divided into five steps. The first three steps are similar to the hydrothermal preparation of porous carbon, with the difference that the temperature is mostly controlled at 180 °C for the hydrothermal preparation of aerogel, while the temperature setting is more flexible for the preparation of porous carbon. The fourth step is to modify the aerogel using a specific solution to obtain special properties such as lipophilicity, hydrophobicity, and high electrical conductivity. The fifth step is the curing of the modified aerogel at low temperature to stabilize its morphology. 

Zhu et al. prepared a structurally simple aerogel using only three steps (Figure 3c) [23]. Firstly, they dried the sliced pomelo peels at 180 °C for 10 h to obtain a sponge-like hydrogel. Then, the hydrogel was freeze-dried at −20 °C for 24 h, followed by vacuum-drying at −80 °C for 48 h to obtain the corresponding PP carbon aerogel precursors. Finally, the PP aerogel precursors was fully carbonized in a high-temperature nitrogen flow to obtain an ultra-lightweight carbon aerogel. This material exhibited significant adsorption performance for oil and various organic solvents on water surfaces. Imran et al. prepared a 3D porous superhydrophobic/superoleophilic carbon aerogel (Figure 3d) [55]. The dried pomelo peels underwent high-pressure hydrothermal treatment at 180 °C for 10 h, followed by freeze-drying at −40 °C for 60 h to obtain a carbon aerogel. The carbon aerogel was then surface-modified with dimethyl siloxane and cured at 120 °C for 3 h to acquire superhydrophobic/superoleophilic properties. Its sufficiently large pore size and superhydrophobic nature allowed it to effectively adsorb emulsified oil from the water surface. Table 4 summarizes the preparation process of typical research of the hydrothermal method to prepare aerogel.

The summaries in Table 3 and Table 4 show that hydrothermal pyrolysis successfully controls the pyrolysis temperature below 300 °C. This is a temperature stage that avoids excessive collapse of PP grapefruit peel cell walls and the formation of abundant micropores compared to the high temperatures required for direct pyrolysis. Table 1 demonstrates that regardless of whether the activation process involves 2 h of activation at 800 °C under N_2_ atmosphere, 1.5 h of activation at 800 °C under N_2_ atmosphere with the addition of KOH, or the utilization of H_3_PO_4_ as an activating agent, the SSA and total pore volume (PV) of the porous carbon nanomaterials prepared through hydrothermal pyrolysis are superior to those prepared through direct pyrolysis.

## 3. Activation

Activation is a critical step after pyrolysis that restores and upgrades the collapsed porous structure during the pyrolysis and is modified with chemical groups on the surface of porous carbon nanomaterials. Although untreated PP has a porous and fluffy structure, the collapse of pores during the carbonization process makes it not advantageous compared to other biomass pyrolysis. In general, the pore collapse is less severe for the porous carbon nanomaterials obtained by hydrothermal pyrolysis due to the relatively low temperature during the process. Regardless of which technology is used to upgrade PP, appropriate activation technology should be selected to obtain porous carbon nanomaterials with ideal performance. 

Activation techniques can be divided into physical activation and chemical activation. Physical activation is the high temperature treatment of pre-carbonized materials under airflow conditions (N_2_, Ar, CO_2_, air, and steam). 

It not only enlarges the narrow pores formed on the surface of the biochar but also generates new pores, thereby increasing the pore volume and SSA of the carbonaceous porous structure [69]. Chemical activation is carried out with the help of chemical substances such as acids (H_2_SO_4_, H_3_PO_4_), alkalis (KOH, NaOH), and salts (ZnCl_2_, K_2_CO_3_, FeCl_3_), which flow between the pores of the pre-carbonized materials for further dehydration, ultimately producing porous carbon nanomaterials with high SSA and PV [70]. Table 1 summarizes the performance of the different activation methods for activating PP. 

### 3.1. Physical Activation 

The most common physical activation method is the pyrolysis of the peel in a high temperature (700 °C to 1000 °C) with N_2_ stream. Although Ar is a more stable inert gas than N_2_, N_2_ provides a higher SSA for the same heating time and is more economical. For the applications in electrodes, supercapacitors, and other materials where SSA is not a key consideration, Ar can be used as a physical activation gas [13,26]. Few studies have used CO_2_ in the activation of PP but the results indicated that CO_2_ activation could provide very limited SSA and PV [71]. However, whichever gas is chosen as the protective gas for physical activation, SSA and PV of the activated materials are limited. In addition, high heating temperatures are required.

### 3.2. Chemical Activation 

The most common chemical activation method is the use of KOH as an activator. This method is inexpensive and requires only a few hours of low temperature heating to activate PP. The result is porous carbon nanomaterials with higher SSA and PV compared to those obtained by physical activation. It is an economical and efficient method. In the preparation of porous carbon, especially, KOH can produce large surface areas and highly microporous structures [71]. H_3_PO_4_ also works well as an activator, but requires a higher activation temperature and longer activation time than KOH. 

### 3.3. Combinational Activation 

However, in many cases, it is difficult to obtain porous carbon nanomaterials with desirable properties in a limited amount of time and energy with only a single activation method. Using a combination of activation technologies to upgrade PP can greatly reduce activation times and obtain materials with higher SSA and PV relative to those materials treated with single activation technology. The most common combination of activated PP carbon precursors is to activate them at high temperature in a N_2_ atmosphere with KOH as the chemical activator [33,34,39]. This method usually allows the preparation of materials with SSA above 1500 m^2^g^−1^ and high PV in less than two hours. The process for treating PP with salts alone as activators is like using KOH, but, generally, the resulting material is moderate in SSA and PV and inferior to that obtained by activation with KOH. Moreover, a combination of various chemical activators can lead to porous carbon nanomaterials with ultra-high SSA and PV. This includes a first step of activation with KOH, followed by a second step of activation with ZnCl_2_ and FeCl_3_ at medium temperature. Compared to the combination of chemical activators and high temperature physical activation mentioned in the previous section, this combination requires a lower activation temperature and the prepared materials tend to have a higher PV [19], but it suffers from difficulty in impurity removal.

In general, there has been a significant amount of research in upgrading PP using a single activation method, but the materials thus obtained have struggled to meet the growing demand for advanced porous carbon nanomaterials in terms of SSA and PV. Physical activation is often present as an aid, driving the chemical activator to maximal effectiveness. The key to exploring efficient activation lies in exploring how to effectively combine multiple chemical activators and to address the attendant problems of complex processes, impurity removal, and material losses.

## 4. Application

Porous carbon nanomaterials derived from PP have large SSA [63], good physicochemical properties [72], and economic feasibility [73], promoting their applications in various fields, including environmental protection, energy storage, and electrochemistry. Specifically, these materials can be utilized as absorbers, batteries, supercapacitors, and catalysts. 

### 4.1. Absorbers

Porous carbon nanomaterials derived from PP are widely applied as absorbers due to their loose and porous structure as well as stable chemical properties. Conventional porous carbon prepared by direct pyrolysis and hydrothermal pyrolysis are suitable for wastewater processing, such as adsorption of heavy metal ions [43,74,75], fluoride [44], ketamine [76], iodate [77], sulfide [78,79], and other pollutants [80], and can also be used as an air purification material to adsorb ammonia gas [81].

**Figure 4:**
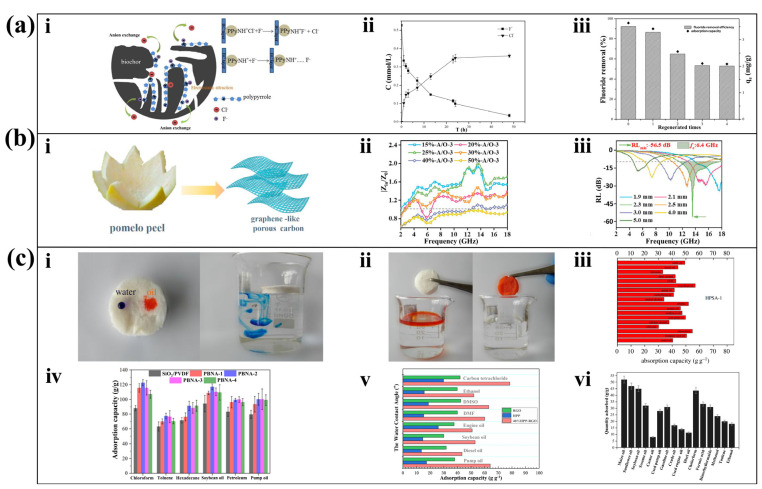
Porous carbon nanomaterials applied as absorbers. (**a**) Fluoride ion adsorption. (**i**). Adsorption process. (**ii**). Change of Cl^−^ and F^−^ concentrations in solution in adsorption process. (**iii**). Regenerative cycle experiment results [44]. (Reproduced with permission, Copyright 2017, Springer Nature, Berlin/Heidelberg, Germany). (**b**) Microwave absorption. (**i**). Graphene-like porous carbon synthesis route. (**ii**). |Z_in_/Z_0_| of samples prepared from different mass fraction HAc-H_2_O_2_ solutions. (**iii**). RL curve at 40% mass fraction of HAc-H_2_O_2_ [85]. (Reproduced with permission, Copyright 2021, Elsevier). (**c**) Oil/organic substance adsorption. (**i**). Hydrophobic sponge aerogel-1 (HPSA-1) in contact with oil/water (water and oil were coloured by methylene blue and Sudan 3, respectively). (**ii**). The carbon aerogel fully absorbs the red oil. The absorption capacities for various oils and organic solvents of (**iii**) HPSA-1 [86]. Reproduced with permission, Copyright 2019, ACS, Washington, DC, USA. (**iv**) Porous biochar/nanofibrous aerogels (PBNA) and the SiO_2_/PVDF aerogel [24]. Reproduced with permission, Copyright 2020, ACS. (**v**) Hydrothermal pomelo peel reduced graphene oxide’s (HPP-RGO) [67]). (Reproduced with permission, Copyright 2021, ACS). (**vi**) 3D porous aerogel [65]. (Reproduced with permission, Copyright 2020, Springer Nature).

Taking the research of polypyridine modified PP [44] as an example, the peel itself owns a fluffy structure and direct pyrolysis treatment makes it rich in attachment sites. However, the porous carbon obtained by direct pyrolysis of PP has a negative charge on its surface and is not suitable for adsorbing anions. The in situ chemical oxidative polymerization method was used to deposit polypyrrole onto the porous carbon, where some of the positively charged nitrogen atoms can attract F^−^ (Figure 4a(i)). Figure 4a(ii) shows that the material can effectively reduce the concentration of F^−^ in the solution. Figure 4a(iii) shows that the material maintains a high adsorption capacity during repeated use testing. However, during ion exchange, the modified material gradually releases Cl^−^ and introduces impurities. To address this problem, magnesium oxide nanoparticles (NPs) can be deposited to achieve F^−^ adsorption as an improvement of deposit polypyrrole. The results of using magnesium oxide-modified porous carbon showed rapid adsorption in wastewater with high F^−^ concentrations without releasing Cl^−^ [82]. Using FeCl_3_ solution to modify the PP can obtain porous carbon with 97.22% decolorization rate of methylene blue solution [83]. Co-pyrolysis of PP and Fe_3_O_4_ powder mixed in graphene suspension can obtain carbon nanocomposites with strong adsorption to ciprofloxacin and sparfloxacin [84]. With the rapid development of nuclear technology, nuclear waste poses a threat to the environment. PP was used as a precursor, immersed in concentrated H_2_SO_4_ solution, and modified with KMnO_4_ to obtain a composite porous carbon with excellent adsorption performance to uranyl [56]. What is more, the role of freeze-drying was highlighted in exploring the adsorption capacity of PP on Congo red. Freeze-dried PP-derived carbon have a stronger adsorption capacity than air-dried and primitive peel derived carbon, which is due to the sublimation of water molecules in freeze-drying that makes PP more porous. At equilibrium concentration, the adsorption capacity of the material increased with the increase in Congo red concentration and temperature [28].

In addition to adsorbing chemical substances, PP-derived porous carbon is lightweight, antioxidant, and has a large SSA, making it a good substrate for electromagnetic absorption materials [87]. However, the lack of magnetic loss capability greatly limits its electromagnetic absorption performance. Hou et al. obtained sparsely structured porous carbon by pyrolysis of PP, with uniform growth of CoFe_2_O_4_ particles in the pores and covered with polyaniline (PANI) coating. CoFe_2_O_4_ particles and polyaniline can function as magnetic loss and dielectric loss, respectively, which gives the composite strong electromagnetic absorption ability [88]. Similarly, celery can also have electromagnetic absorption capacity through direct pyrolysis carbonization with Fe-Co deposition [89]. Zhao et al. treated the precursors with HAc-H_2_O_2_ solution after the hydrothermal pyrolysis PP step, where HAc reduced the thickness of the nanosheets and H_2_O_2_ increased the porosity. The obtained porous carbon had a graphene-like structure (Figure 4b(i)). Materials treated with 10% and 40% concentration of H_2_O_2_ are required to have an impedance matching of |Z_in_/Z_0_| close to 1 (Figure 4b(ii)), indicating that all incident electromagnetic microwaves penetrate the interior of the material, which is favorable for subsequent electromagnetic wave attenuation. Figure 4b(iii) shows that the material has good absorption of microwaves with a bandwidth from 1.9 mm to 5 mm, and the most obvious absorption of microwaves at 2.3 mm [85].

Carbon aerogel owns ultra-low density so it can easily float on the water surface, which makes it ideal for adsorbing oil and organic solvents [90]. Shi et al. prepared a sponge aerogel by high-speed dispersion, freeze-drying, and methyltrimethoxysilylation. The aerogel owned a high selectivity for oil and water (Figure 4c(i)) and a superhydrophobic surface that can easily absorb oil (Figure 4c(ii)). In further adsorption tests on typical oils and organics, the aerogel showed significant adsorption on soybean oil and dimethyl sulfoxide (DMSO) and chloroform (Figure 4c(iii)) [86]. Chen et al. treated the PP precursor prepared by hydrothermal method with poly vinylidene fluoride (PVDF)/dimethylformamide (DMF) solution to obtain nanofiber aerogel, which has ultra-high adsorption capacity for soybean oil, chloroform, and pump oil (Figure 4c(iv)) [24]. Using hydrothermally pretreated pomelo peel (HPP) and reduced graphene oxide (RGO) as raw materials, graphene aerogel can be prepared by a two-step hydrothermal method. The whole preparation process is green and environmentally friendly, and the obtained materials are effective in adsorption of carbon tetrachloride and various oils (Figure 4c(v)) [67]. With a simple hydrothermal and silanization treatment of PP, carbon aerogels with strong adsorption capacity for a variety of oils and organic solvents can be obtained (Figure 4c(vi)) [65]. In addition, recent research has found that aerogel prepared by hydrothermal PP can be used to absorb heavy metal ions [91], pigments [92] and fluoroquinolone antibiotic [93].

### 4.2. Battery

**Figure 5:**
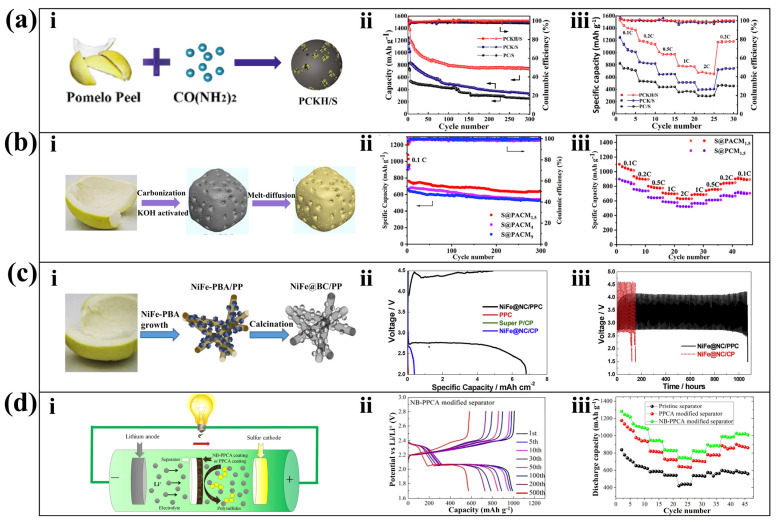
Porous carbon nanomaterials applied as battery electrode and separator. (**a**) Sulfur host for Li-S batteries. (**i**) Streamlined preparation process, (**ii**) cycling performance, and (**iii**) cell rate performance [94]. (Reproduced with permission, Copyright 2020, Elsevier). (**b**) Cathode of Li-S battery, (**i**) streamlined preparation process, (**ii**) cycling performance, and (**iii**) cathodic rate capability [95]. (Reproduced with permission, Copyright 2022, Springer Nature). (**c**) Free-standing cathode for Li-CO_2_ batteries, (**i**) streamlined preparation process, (**ii**) charging and discharging performance and (**iii**) voltage variation under long time charging and discharging [96]. (Reproduced with permission, Copyright 2019, Elsevier). (**d**) Li-S battery separator, (**i**) working principle, (**ii**) the charging and discharging curves, and (**iii**) discharge capacity of the battery after the adoption of the separator [97]. (Reproduced with permission, Copyright 2019, Elsevier).

Porous carbon nanomaterials prepared by direct and hydrothermal pyrolyzing the PP method can also be used for battery electrodes and battery separators. Currently, the most extensive research attention has been paid to the application of PP-derived porous carbon nanomaterials in Li-S battery electrodes. The abundant porosity of the porous carbon provides excellent physical constraints for S, accelerating the transfer of electrons and ions. Xiao et al. obtained nitrogen-doped carbon from PP treated with urea by pyrolysis (Figure 5a(i)). Nitrogen doping provides chemical constraints for S in addition to physical constraints, limiting the shuttle behavior of polysulfides. The KOH-activated carbon materials can maintain a Coulombic efficiency of over 98% after 300 cycles (Figure 5a(ii)) and has high current rate capability (Figure 5a(iii)) [94]. Ma et al. activated directly pyrolyzed PP with KOH and directly prepared an S-rich Li-S battery cathode with sulfur (Figure 5b(i)). The material can accommodate 9 times its weight of S and exhibit strong chemical bonding with lithium polysulfides. After 300 cycles at 1 C, the material still has a high specific discharge capacity of 636.9 mAh g^−1^ (Figure 5b(ii)), and the cathode shows excellent discharge performance at different current densities (Figure 5b(iii)) [95].

In addition, PP-derived porous carbon nanomaterials can also be used for Li-CO_2_ batteries. Liang et al. first synthesized NiFe-prussian blue analog/PP precursor by co-precipitation [98], and then treated the precursor with Na_3_C_6_H_5_O_7_·2H_2_O and K_3_[Fe(CN)_6_] to obtain NiFe@NC/PPC (Figure 5c(i)). This material not only retains the three-dimensional porous characteristics of biomass, but also the NiFe@NC attached to its surface can catalyze the cathode reaction of Li-CO_2_ batteries. The stable discharge plateau of the NiFe@NC/PPC cathode battery is around 2.76 V, with an overpotential of approximately 0.04 V (Figure 5c(ii)), and it exhibits a perfect Coulombic efficiency of 72.0%, which is superior to similar products. Compared to using carbon paper (CP) as the substrate for NiFe@NC attachment, using PP derived carbon (PPC) can achieve higher cycling stability (Figure 5c(iii)) [96]. 

Moreover, porous carbon nanomaterials derived from PP can also be used for seawater batteries [99]. Using a simple hydrothermal treatment and NaOH activation, porous carbon with defects and self-doped oxygen vacancies can be formed. This material can efficiently catalyze oxygen evolution/reduction reactions and is cost-effective, making it suitable for large-scale applications in seawater batteries. By uniformly mixing the hydrothermal PP precursor with NaBiO_3_ and co-firing, a biochar and metal bismuth composite material can be produced. It can be applied as the negative electrode in vanadium redox flow batteries, efficiently catalyzing the V^3+^/V^2+^ oxidation-reduction reaction, reducing the polarization of the vanadium battery, and increasing the energy density of the battery [100].

Some studies have also focused on the use of PP-derived porous carbon nanomaterials as battery separator. To reduce the shuttle effect of lithium polysulfide between sulfur cathode and lithium anode in Li-S cells, nitrogen and boron dual-doped carbon aerogel (NB-PPCA) prepared by hydrothermal method and NH_4_HB_4_O_7_ treatment was used as coat on the separator in modified Li-S cells (Figure 5d(i)). It can not only reduce the charge transfer resistance inside the battery but can also immobilize the soluble polysulfide by adsorption, which facilitates the reuse of the active material in the charge/discharge cycle. In the cyclic voltammetry test, the Li-S battery with NB-PPCA as the separator still has a high capacity after 500 cycles (Figure 5d(ii)). Figure 5d(iii) shows that the rate of Li-S cells with NB-PPCA separator is significantly higher than that of those with PPCA separator and those with pristine separator [97].

### 4.3. Supercapacitor

**Figure 6:**
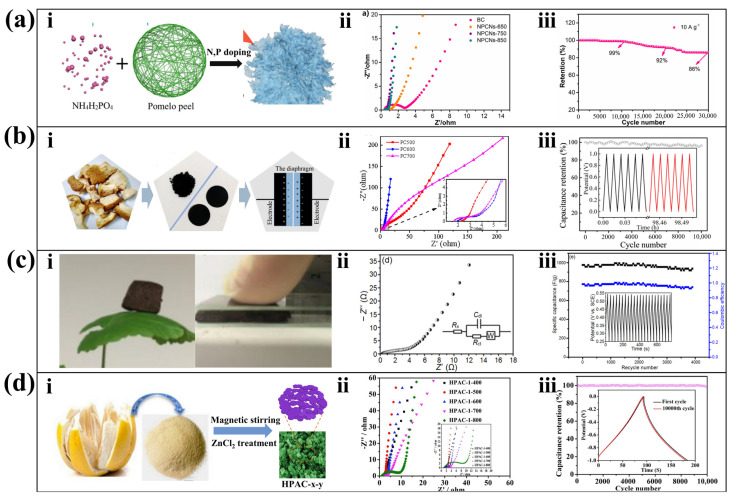
Porous carbon nanomaterials applied as supercapacitor. (**a**) N, P co-doped porous carbon supercapacitor: (**i**) streamlined preparation process, (**ii**) Nyquist plot, and (**iii**) cycling performance at a current density of 10 Ag^−1^ [29]. (Reproduced with permission, Copyright 2022, Elsevier). (**b**) High nitrogen content carbon supercapacitor: (**i**) streamlined preparation process, (**ii**) Nyquist plot and (**iii**) the first six (black line), and last six (red line) cycles stability of the supercapacitor [103]. (Reproduced with permission, Copyright 2018, Springer Nature). (**c**) Cobalt nickel aluminum layered double (CoNiAl-LDH) carbon aerogel: (**i**) demonstration of light weight and flexibility features, (**ii**) Nyquist plot, and (**iii**) the cycling performance (the inset is the charge/discharge curves) [64]. (Reproduced with permission, Copyright 2017, Elsevier). (**d**) Hemicellulose-derived porous activated carbon (HPAC) supercapacitor: (**i**) streamlined preparation process, (**ii**) Nyquist plot, and (**iii**) the cycling performance of HPAC (the inset is galvanostatic charge-discharge curves of the first cycle and the 10,000th cycle) [104]. (Reproduced with permission, Copyright 2020, Elsevier).

Porous carbon obtained by pyrolyze biomass is an ideal material for supercapacitors [101]. The surface layer of porous carbon can be modified with oxygen-containing functional groups [20] and nitrogen-sulfur dopants by direct pyrolysis of PP [102]. Li et al. mixed pomelo peel with NH_4_H_2_PO_4_ and used direct pyrolysis technology to prepare N, P co-doped hierarchical porous carbon nanosheets (Figure 6a(i)). It has a large SSA, abundant defects, and active sites, thus exhibiting strong electrochemical energy storage performance. Among them, the Nernst plot of the porous carbon nanosheets prepared at 750 °C had the largest slope in the low-frequency region (Figure 6a(ii)), indicating the smallest ion diffusion resistance and the lowest electrochemical impedance. Its storage capacity was excellent (314 ± 2.6 Fg^−1^). The capacitance retention rate was 99% after 10,000 cycles, and still remained at 86% after 30,000 cycles (Figure 6a(iii)) [29]. Fu et al. carbonized PP in a nitrogen atmosphere and activated the carbonized PP by mixing it with KOH (Figure 6b(i)). The porous carbon obtained at this temperature has a distinct stratified pore structure and is more suitable for application in supercapacitors. Figure 6b(ii) shows that the Nernst plot of the material prepared by carbonization at 700 °C is the most vertical in the low-frequency region, indicating the highest capacitance. However, the high-frequency region of the Nernst plot (inserted part) shows that the material prepared by carbonization at 600 °C has the largest slope, indicating that the diffusion resistance of electrolyte ions inside this material is low during the charging and discharging process and the diffusion path is short. The Figure 6b(iii) shows that the supercapacitor prepared at 600 °C has good capacitance capacity and cycling stability with a specific capacity retention rate of 96.2% after 10,000 cycles [103].

Porous carbon nanomaterials obtained from hydrothermal pyrolysis of PP are more suitable for the preparation of supercapacitors than porous carbon obtained by direct pyrolysis. This is because the hydrothermal brings more oxygen-containing functional groups to the porous carbon while reducing impurities and pore collapse. Liu et al. prepared porous carbon with a three-dimensional hierarchical and interconnected honeycomb structure by hydrothermal pyrolysis combined with KOH activation process [27]. Using a similar strategy, Qu et al. used 10% concentration of ammonia to replace deionized water as a solvent to increase the nitrogen doping concentration of porous carbon. This significantly enhanced the electrical capacity of the porous carbon nanomaterials [33]. Replacing deionized water with H_3_PO_4_ solution can produce P-doped porous carbon, which has a high electric capacity, similar to N-doped porous carbon [31]. Zhang et al. prepared a carbon aerogel by hydrothermal method, and then treated it with Co(NO_3_)_2_·6H_2_O, Ni(NO_3_)_2_·6H_2_O, and Al(NO_3_)_3_·9H_2_O to obtain CoNiAl-LDH@CA nanocomposites, showing characteristics of light weight and ultra-high flexibility (Figure 6c(i)). The material exhibited a steep Nernst plot in the tests (Figure 6c(ii)) and the capacitance could still be maintained at around 96% after 4000 cycles (Figure 6c(iii)) [64]. Additionally, instead of using PP directly as a carbon source, the high carbon content of hemicellulose in PP can be extracted as a substitute. The pomelo peel was treated with HCl/ethanol and centrifuged to obtain hemicellulose. Next, activation was performed using ZnCl_2_. ZnCl_2_ reacts with OH groups to dissolve hemicellulose and provide a backbone for porous carbon (Figure 6d(i)). Among the materials obtained by activation at different temperatures, the Nernst plot curve of the material activated under 500 °C conditions are closest to vertical (Figure 6d(ii)), confirming that the capacitance is superior to other samples. The capacitance retention rate of the material after 10,000 cycles is about 98.6%, and the constant current charge-discharge curves of the 1st and 10,000th cycles also show excellent cycling stability (Figure 6d(iii)) [104].

### 4.4. Catalyst

**Figure 7:**
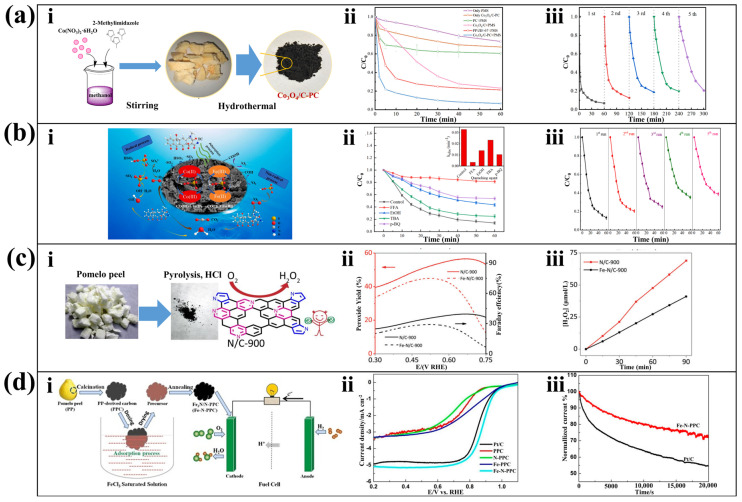
Porous carbon nanomaterials applied as catalyst. (**a**) Co_3_O_4_/PP composite carbon activating PMS to degrade CIP: (**i**) streamlined preparation process, (**ii**) catalytic performance of Co_3_O_4_/C-PC catalysts for CIP, and (**iii**) Co_3_O_4_/PP reusability test results [93]. (Reproduced with permission, Copyright 2020, Elsevier). (**b**) Catalytic degradation of TC by PP: (**i**) reaction principle, (**ii**) test for the main types of reactive radicals in catalytic systems, and (**iii**) Co-Fe@PPBC reusability test results [94]. (Reproduced with permission, Copyright 2022, Elsevier). (**c**) N/C catalyst for catalytic H_2_O_2_ production: (**i**) streamlined preparation process, (**ii**) H_2_O_2_ productivity and Faradaic efficiency of N/C-900 and Fe-N/C-900, and (**iii**) plot of the amount of H_2_O_2_ produced by their catalytic reaction versus time [95]. (Reproduced with permission, Copyright 2017, The Society). (**d**) In situ anchoring Fe_2_N nanoparticles on nitrogen-doped PP-derived carbon to enhanced oxygen reduction reaction: (**i**) preparation and workflow, (**ii**) linear scanning voltammetry (LSV) curves, and (**iii**) results of long-term stability tests in KOH solution [96]. (Reproduced under the terms of the CC-BY 4.0 Creative Commons Attribution License, Copyright 2017, The Authors, published by MDPI, Basel, Switzerland).

Given that porous carbon is chemically stable and does not easily react with acids and bases, it is also a suitable material for catalyst matrices [105,106]. Porous carbon nanomaterials derived from PP have been widely applied for catalyzing the decomposition of chemical substances in wastewater. Fe@pomelo-derived carbon can be prepared by hydrothermal pyrolysis of a mixture of PP powder and FeCl_3_·6H_2_O solution, and it can be used for catalyzing the degradation of p-nitrophenol in wastewater [107]. SMX, a bacteriostatic antibiotic, is widely distributed in pharmaceutical and livestock wastewater. Nitrogen-doped PP carbon treated with NaHCO_3_ can be used as an oxidation catalyst for the oxidation of SMX [42]. Metal-organic framework (MOFs) derived metal oxide/carbon catalysts have been widely studied in the field of environmental remediation. However, their support and aggregation have always been a challenge. Zhang and Dai utilized the strong water absorption ability of dried PP to fully absorb zeolitic imidazole framework-67 (ZIF-67) and co-thermally decompose it to obtain PP/ZIF-67 derivative composite material (Co_3_O_4_/C-PC) (Figure 7a(i)). It can efficiently activate peroxymonosulfate (PMS) to degrade ciprofloxacin (CIP) (Figure 7a(ii)) and maintain strong activation ability after multiple repetitions (Figure 7a(iii)) [108]. To address the threat of widespread use of antibiotics on the environment, a Co-Fe@ pomelo peel biochar composite (PPBC) composite material was proposed for the degradation of tetracycline (TC), which is extensively used in animal and human medicine and is non-degradable under natural conditions. The material utilizes electron transfer between Co^2+^ and Fe^3+^ to initiate the decomposition of PMS to generate SO^4-^, OH, and activated oxygen, which can promote TC decomposition. Figure 7b(ii) shows that the addition of ethanol (EtOH) and tert-butyl alcohol (TBA) has a significant inhibitory effect on TC removal, as these chemicals can efficiently remove SO^4-^ and ∙OH, demonstrating that these two ions play a key role in TC degradation. During the process of repeated reuse, Co-Fe@PPBC also shows stable catalytic effects, demonstrating the feasibility of its recycling (Figure 7b(iii)) [109].

Meanwhile, PP-derived porous carbon nanomaterials can also be used for catalytic production of chemical products. H_2_O_2_ is in great demand in industry, and the vast majority of H_2_O_2_ is produced by the anthraquinone process. The limitation of this indirect batch process is that the continuous addition of anthraquinone causes great harm to the ecosystem. In addition, this method requires the extraction of H_2_O_2_ from solvents, which can easily cause explosions. To address these issues, a nano-porous N/C electrocatalyst has been proposed for efficiently producing H_2_O_2_ by catalyzing the redox reaction of O_2_ and H^+^. The material is prepared by direct pyrolysis and activated by HCl treatment (Figure 7c(i)). Although transition metals such as Fe can improve the graphitization degree of PP during pyrolysis, the material prepared without Fe has higher selectivity for H_2_O_2_ in the actual activation process (Figure 7c(ii)), and higher efficiency in producing H_2_O_2_ (Figure 7c(iii)) [110]. The co-pyrolysis of PP powder immersed in Fe(NO_3_)_3_ solution can produce porous carbon loaded with Fe_3_O_4_ particles. This material can be used as a catalyst for the hydrogenation of CO_2_ to light olefins, addressing the issues of environmental unfriendliness and high cost associated with traditional methods of CO_2_ hydrogenation to light olefins [111]. Mixing the hydrogel precursor obtained from hydrothermal treatment of PP with a Ni(NO_3_)_2_·6H_2_O solution, followed by complete carbonization under a high-temperature nitrogen atmosphere, leads to the production of Ni-loaded PP carbon aerogels. This material can efficiently catalyze the ethanol cracking reaction in biomass waste, enabling the large-scale and economical production of hydrogen gas [112].

In addition to the above applications, porous carbon nanomaterials prepared by pyrolysis of PP can be used for catalytic electrode reactions. Wang et al. anchored Fe_2_N nanoparticles in situ onto PP-derived carbon for highly efficient catalysis of redox reactions in alkaline medium. The Fe-based nanocrystals, composed of Fe_2_N and other phases, were loaded onto the pomelo peel-derived carbon (N-PPC) previously impregnated with FeCl_3_·6H_2_O solution, followed by calcination in NH_3_ atmosphere (Figure 7d(i)). The resulting material was rich in Fe-N-C bonding catalytic sites and directly catalyzed the four-electron redox reactions. Comparative experiments showed that the initial potential of the redox reactions in the system using Fe-N-PPC was higher than those using Pt/C, N-PPC and other catalysts, indicating better catalytic performance (Figure 7d(ii)). Moreover, the system using the material exhibited a higher cathodic current rate than that using Pt/C during a reaction period of 20,000 s, demonstrating good catalytic stability (Figure 7d(iii)) [113].

## 5. Discussion

The carbon nanomaterials prepared by pyrolysis of PP have a wide application space. Moreover, the following issues need to be considered for PP synthesis lines:

(1) Environmentally friendly throughout the process. Considering the overall environmental impact throughout the synthesis, application, and final disposal of porous carbon nanomaterials, various factors need to be considered. During the synthesis stage, using waste PP as the raw material, direct pyrolysis only requires a protective gas and high temperature, while hydrothermal pyrolysis typically involves an acidic or alkaline liquid environment. The activation process can be achieved through physical activation with a protective gas or chemical activation using acids, alkalis, or salts as activating agents. Subsequently, impurity removal may require cleaning the product with an HCl solution. Thus, appropriate treatment and recycling should be carried out for acidic and alkaline waste liquids, as well as solutions containing salts such as FeCl_3_ and ZnCl_2_. Additionally, during the synthesis process, in addition to solid products, there may be accompanying liquid products (tar) and gaseous products (methane, CO) [114]. Proper collection of these byproducts not only reduces environmental pollution but also yields valuable secondary products. During the application stage, porous carbon nanomaterials have simple combinations and stable physicochemical properties that do not pose a risk of pollution to the environment. They can be employed in adsorbers and catalysts to address environmental pollution issues. In the final disposal stage, porous carbon nanomaterials that have not adsorbed chemical substances can be reused, incinerated, or buried without adverse effects on the environment. However, activated carbon that has adsorbed metal ions, fluoride ions, and other chemical substances should undergo desorption before being recycled or subjected to incineration or burial.

(2) Expandability of the production line. The theoretical analysis of synthesizing porous carbon materials from PP suggests that direct pyrolysis generally requires a protective gas atmosphere, a heating range of 600 °C to 900 °C, and a duration of 1 to 3 h. Hydrothermal pyrolysis, on the other hand, typically necessitates a high-pressure reactor, a heating range of 150 °C to 300 °C, and a duration of 6 to 24 h. Activation is performed using the most effective combinational activation method, which involves mixing the pyrolyzed product with KOH under a N_2_ atmosphere, heating it within the range of 700 °C to 800 °C, and maintaining it for a duration of 1 to 2 h. In addition to being suitable for the synthesis of porous carbon nanomaterials from PP, the technical specifications can also be applied to process other abundant waste biomass, such as banana peels [115], orange peels [116], coconut shells [117], cornstalk [118], and pomegranate peels [119].

(3) Energy consumption issues. In comparing direct pyrolysis and hydrothermal pyrolysis, it is observed that direct pyrolysis necessitates a high-temperature and flowing gas environment, whereas hydrothermal pyrolysis, which operates at lower temperatures and in a closed gas environment, consumes comparatively less energy. Microwave-assisted hydrothermal pyrolysis technology requires more energy than conventional hydrothermal pyrolysis technology, which is because the microwave absorption capacity of PP is not good, and a lot of energy will be lost during the microwave heating process [120]. When considering physical activation and chemical activation techniques, the utilization of suitable chemical activating agents can eliminate the need for or substantially reduce the high-temperature heating duration required for physical activation, thereby effectively reducing overall energy consumption.

Despite the significant advancement of synthesis of porous carbon nanomaterials from PP, there are still some challenges. We have summarized these challenges and provided potential solutions:

(1) The production process is complex. Direct pyrolysis requires high temperatures and often results in suboptimal physical properties of the product. Traditional and microwave-assisted hydrothermal pyrolysis do not require high temperatures but require complex high-pressure heating equipment, limiting scale-up production. In particular, microwave-assisted hydrothermal pyrolysis lacks high-temperature and high-pressure microwave reactors with sufficient capacity and precise control of heating rate and temperature maintenance [121]. Therefore, it is necessary to develop suitable chemical reaction equipment, including but not limited to reactors with large capacity, sealed and adjustable temperature and pressure, and high-temperature and high-pressure microwave reactors capable of precise control of microwave power, microwave variation rate, and temperature maintenance ability. In addition, a simple, atmospheric pressure, and low-temperature pyrolysis route for PP can be explored, such as salt melt pyrolysis, a common biomass pyrolysis technology. Low-melting-point salts provide a good solid-phase reaction environment for the carbonization process. Salt acts as a dehydrating agent, promotes the decomposition of carbonaceous materials during pyrolysis, and limits the formation of tar. Salt melt pyrolysis technology has the advantages of a low operating temperature [122], short reaction time [123], and simple equipment [124]. It is also possible to use laser-induced graphene (LIG) technology [125] to create graphene monolayer structures on the surface of PP by laser heating. This method has the advantages of no need for high temperature and pressure, fast speed, and simple process [126]. The use of this technology to process PP has the potential to produce electrodes with excellent conductivity [127] or highly sensitive sensors [128].

(2) Mechanisms are not very clear. The hydrothermal pyrolysis route produces materials with good properties. However, it has a long carbonization time. Existing research has not fully understood the heat and mass transfer mechanisms within the porous biomass structure in the hydrothermal reactor. Additionally, there is a lack of data on controlling the continuous operation of the reactor under high pressure conditions [129]. In this situation, the designed assembly line has a single function and is unable to process other types of biomasses, and its stability is questionable. Therefore, the microscopic reaction mechanism of biomass hydrothermal pyrolysis needs to be further revealed to provide theoretical support for the design of flexible production chains with adjustable reaction parameters. At the same time, there is a need to design sensors to monitor the hydrothermal pyrolysis process inside high-pressure reactors, which can continuously monitor the operation data of the reactor under high-pressure conditions.

## 6. Conclusions and Outlook

In this review, we have systematically summarized research of upgrading PP into porous carbon nanomaterials. In the first section, we discussed two pyrolysis technologies, direct pyrolysis and hydrothermal pyrolysis. Direct pyrolysis has the advantages of a mature technological process, simple operation, and ease of large-scale manufacturing. However, it also has disadvantages, such as low surface area of the product, high energy consumption, and high activation technology requirements. Hydrothermal pyrolysis can be used to upgrade PP into porous carbon and aerogel. This technology has advantages, such as low reaction temperature and higher SSA product compared to direct pyrolysis. However, it also has disadvantages, including long reaction time, complex operation, and high-pressure equipment requirements. Microwave-assisted pyrolysis, as an extension of hydrothermal technology, not only carbonizes PP in a more rapid manner but also removes tar from the pores, providing porous carbon nanomaterials with higher SSA. In the second section, we discuss activation techniques for the produced porous carbon nanomaterials, including physical activation, chemical activation, and their combination. The physical activation process is simple and time-saving, usually using N_2_ as a protective gas. However, the SSA and PV of its products are difficult to meet the high-performance requirements of porous carbon nanomaterials, and maintaining a high-temperature environment consumes a large amount of energy. Chemical activation, on the other hand, has higher efficiency and lower energy consumption. However, it often faces the challenge of long activation time. Combining physical activation and chemical activation can result in porous carbon nanomaterials with high SSA and PV in a shorter time. In the third section, we summarized the applications of PP-derived porous carbon nanomaterials, including adsorbers, batteries, supercapacitors, and catalysts. Adsorbers are the widest applications, particularly for the adsorption of oil and organic solvents from water surfaces, which is facilitated by using aerogels prepared by hydrothermal pyrolysis. By doping N, S, and metal ions into the porous carbon materials, they exhibit excellent electrochemical performance and can be used to prepare battery electrodes, battery separators, and supercapacitors. By treating the porous carbon materials with metal compound solutions, diverse functional catalysts can be prepared to efficiently catalyze the decomposition of chemical substances in wastewater, chemical production, and redox reactions in batteries. 

## Figures and Tables

**Table 1:** Summary of the properties and activation conditions of porous carbon nanomaterials derived from PP using different pyrolysis and activation methods.

Method		Reagent	SSA (m^2^g^−1^)	PV (cm^3^g^−1^)	Time (h)	Temp (°C)	Ref.
D ^1^	Physical	Ar	158 m^2^g^−1^	-	2 h	1000 °C	[13]
	Physical	N_2_	809.2 m^2^g^−1^	-	2 h	800 °C	[14]
	Physical	N_2_	807.7 m^2^g^−1^	0.43 cm^3^g^−1^	2 h	900 °C	[15]
	Chemical	KOH	1892.1 m^2^g^−1^	1.09 cm^3^g^−1^	4 h	80 °C	[16]
	Chemical	H_3_PO_4_	1272 m^2^g^−1^	1.85 cm^3^g^−1^	24 h	-	[17]
	Chemical	FeCl_3_	26.48 m^2^g^−1^	0.14 cm^3^g^−1^	2 h	-	[18]
	Combinational	KOH + ZnCl_2_ + FeCl_3_	2463 m^2^g^−1^	2.05 cm^3^g^−1^	2 h	550 °C	[19]
	Combinational	KOH/Ar	2630 m^2^g^−1^	-	72 h/2 h	-/800 °C	[20]
	Combinational	KOH + N_2_	1796 m^2^g^−1^	1.48 cm^3^g^−1^	1.5 h	800 °C	[21]
	Combinational	KOH + N_2_	2457 m^2^g^−1^	1.14 cm^3^g^−1^	2 h	600 °C	[22]
H ^1^	Physical	N_2_	759.7 m^2^g^−1^	0.45 cm^3^g^−1^	1 h	800 °C	[23]
	Physical	N_2_	838 m^2^g^−1^	0.85 cm^3^g^−1^	2 h	800 °C	[24]
	Physical	N_2_	10.9 m^2^g^−1^	-	1 h	800 °C	[25]
	Physical	Ar	232.4 m^2^g^−1^	0.26 cm^3^g^−1^	2 h	1000 °C	[26]
	Chemical	KOH	832 m^2^g^−1^	-	12 h	-	[27]
	Chemical	KOH	4.0 m^2^g^−1^	-	2 h	200 °C	[28]
	Chemical	NH_4_H_2_PO_4_	908.2 m^2^g^−1^	0.82 cm^3^g^−1^	3 h	750 °C	[29]
	Chemical	KCl	296 m^2^g^−1^	0.3 cm^3^g^−1^	1 h	900 °C	[30]
	Chemical	H_3_PO_4_	1432.1 m^2^g^−1^	0.72 cm^3^g^−1^	3 h	120 °C	[31]
	Combinational	K_2_C_2_O_4_ + N_2_	785.1 m^2^g^−1^	0.53 cm^3^g^−1^	0.5 h	750 °C	[32]
	Combinational	KOH/N_2_	1727.7 m^2^g^−1^	1.00 cm^3^g^−1^	2 h	800 °C	[33]
	Combinational	KOH + N_2_	2504 m^2^g^−1^	1.19 cm^3^g^−1^	1.5 h	800 °C	[34]
	Combinational	ZnCl_2_/ZnCl_2_ + Ar_2_	1582 m^2^g^−1^	-	4 h/2 h	-/800 °C	[35]
	Combinational	KHCO_3_/N_2_	1146.2 m^2^g^−1^	-	2 h	800 °C	[36]
	Combinational	KOH + Ar	2191 m^2^g^−1^	1.03 cm^3^g^−1^	1 h	800 °C	[37]
	Combinational	KOH + N_2_	904.1 m^2^g^−1^	0.51 cm^3^g^−1^	2 h	700 °C	[38]
	Combinational	KOH/KOH + N_2_	1870 m^2^g^−1^	0.99 cm^3^g^−1^	24 h/1 h	-/800 °C	[39]

^1^ D means direct pyrolysis and H means hydrothermal pyrolysis.

**Table 2:** Summary of typical porous carbon preparation processes with direct pyrolysis method.

Pre-Process	Carbonize	Activation	Ref.
Dice, clean, and dry at 60 °C, then treat with K_2_FeO_4_	600 °C for 1 h, under N_2_ flow	-	[43]
Dice and dry at 80 °C for 24 h	450 °C, under N_2_ flow	KOH	[16]
Dice and dry at 80 °C for 24 h	550 °C for 2 h, under Ar flow	KOH/ZnCl_2_/FeCl_3_	[19]
Dice, clean, and dry at 60 °C for 48 h	600 °C for 1 h	FeCl_3_	[44]
Dice and dry at 60 °C for 30 h	1000 °C for 2 h, under Ar flow	Ar	[13]
Dice	800 °C for 2 h, under Ar flow	KOH/Ar	[20]
Dice and vacuum freeze dry for two days	700 °C for 2 h, under N_2_ flow	H_3_PO_4_	[17]
Dice, clean, and dry at 70 °C for 24 h	900 °C for 3 h, under Ar flow	-	[45]
Dice, clean, and dry at 100 °C for 12 h	800 °C for 1 h, under Ar flow	KOH	[46]

**Table 3:** Summary of typical conventional porous carbon preparation processes using hydrothermal pyrolysis method.

Pre-Process	High-Pressure Carbonize	Activation	Freeze-Dry	Ref.
Dice and dry at 60 °C for 12 h	Citric acid mix, 200 °C for 6 h	KOH/N_2_	Dry for 48 h	[27]
Dice and dry at 60 °C	Water mix, 200 °C for 24 h	H_2_SO_4_/KMnO_4_	60 °C for a night	[56]
Dice and dry	Co-heat with KOH solution, 200 °C for 2 h	KOH	Dry	[28]
Clean, crush, and dry	180 °C for 15 h	K_2_C_2_O_4_/N_2_	Dry	[32]
Dice and dry	160 °C for 12 h, then 300 °C for 1.5 h in Muffle furnace	KOH/N_2_	-	[33]
Dice and dry	160 °C for 6 h	H_3_PO_4_/Ar	Dry	[31]

**Table 4:** Summary of typical aerogel preparation processes with hydrothermal method.

Pre-Process	High-Pressure Carbonize	Freeze-Dry	Modify	Solidify	Ref.
Clean, dice and dry	180 °C for 10 h	−40 °C for 60 h	Dimethyl siloxane treatment	120 °C for 3 h	[55]
Dice and dry	180 °C for 6.5 h	−40 °C for 48 h	Urea and anhydrous methanol treatment	150 °C for 12 h	[64]
Dice	180 °C for 10 h	−80 °C for 48 h	Dimethicone and melamine Treatment	120 °C for 12 h	[65]
Dice	180 °C for 10 h	Dry	Add SiO_2_ nanofibers, use homogenizer for treatment	80 °C for 6 h	[24]
Clean, dice and dry	100 °C for 10 h	−18 °C for 24 h	Silanization treatment	-	[66]
Clean, dice and dry	180 °C for 12 h	Dry	-	-	[67]
Dice and dry	180 °C for 12 h	Dry	-	-	[68]

## Data Availability

Not applicable.
